# Distribution of Human Norovirus in the Coastal Waters of South Korea

**DOI:** 10.1371/journal.pone.0163800

**Published:** 2016-09-28

**Authors:** Man Su Kim, Eung Seo Koo, Yong Seon Choi, Ji Young Kim, Chang Hoon Yoo, Hyun Jin Yoon, Tae-Ok Kim, Hyun Bae Choi, Ji Hoon Kim, Jong Deok Choi, Kwon-Sam Park, Yongsik Shin, Young-Mog Kim, GwangPyo Ko, Yong Seok Jeong

**Affiliations:** 1 Department of Biology and Research Institute of Basic Sciences, Kyung Hee University, Seoul, South Korea; 2 Department of Seafood Science and Technology, Institute of Marine Industry, Gyeongsang National University, Tongyeong, Gyeongnam, South Korea; 3 Department of Food Science and Biotechnology, College of Ocean Science and Technology, Kunsan National University, Kunsan, South Korea; 4 Department of Environmental Engineering & Biotechnology, Mokpo National Maritime University, Mokpo, South Korea; 5 Department of Food Science and Technology, Pukyong National University, Busan, South Korea; 6 Department of Environmental Health Sciences, Graduate School of Public Health, Seoul National University, Seoul, South Korea; Australian National University, AUSTRALIA

## Abstract

The presence of human norovirus in the aquatic environment can cause outbreaks related to recreational activities and the consumption of norovirus-contaminated clams. In this study, we investigated the prevalence of norovirus genogroups I (GI) and II (GII) in the coastal aquatic environment in South Korea (March 2014 to February 2015). A total of 504 water samples were collected periodically from four coastal areas (total sites = 63), of which 44 sites were in estuaries (clam fisheries) and 19 were in inflow streams. RT-PCR analysis targeting ORF2 region C revealed that 20.6% of the water samples were contaminated by GI (13.3%) or GII (16.6%). The prevalence of human norovirus was higher in winter/spring than in summer/fall, and higher in inflow streams (50.0%) than in estuaries (7.9%). A total of 229 human norovirus sequences were identified from the water samples, and phylogenetic analysis showed that the sequences clustered into eight GI genotypes (GI.1, 2, 3, 4, 5, 6, 7, and 9) and nine GII genotypes (GII.2, 3, 4, 5, 6, 11, 13, 17, and 21). This study highlighted three issues: 1) a strong correlation between norovirus contamination via inflow streams and coastal areas used in clam fisheries; 2) increased prevalence of certain non-GII.4 genotypes, exceeding that of the GII.4 pandemic variants; 3) seasonal shifts in the dominant genotypes of both GI and GII.

## Introduction

Acute gastroenteritis causes the second greatest burden of all infectious diseases, estimated at 89.5 million disability-adjusted life-years and 1.45 million deaths worldwide every year [1]. In particular, human norovirus (HNoV) has been reported as the major cause of non-bacterial acute gastroenteritis in patients of all ages, responsible for approximately 90% of all outbreaks of viral gastroenteritis in the world [2–6]. HNoV can infect via multiple routes, and is transmitted through contact with gastroenteric effluents originating from infected individuals [7]. At least 70% of outbreaks have occurred in semi-closed communities [8–10].

Noroviruses (NoVs) are small non-enveloped viruses in the Caliciviridae family with a positive single-stranded RNA genome of 7.5–7.7 kb in length, which is organized into three or four open reading frames (ORFs) [11–14]. ORF1 encodes six non-structural proteins, including the viral RNA-dependent RNA polymerase, while ORF2 and ORF3 encode the major (VP1) and minor (VP2) capsid proteins, respectively [15]. After a viral incubation period of 12 hours to 2 days, a patient generally experiences acute symptoms, such as vomiting, diarrhea, nausea, abdominal cramps, and low-grade fever [10]; immunocompromised patients are susceptible to chronic gastroenteritis [16]. NoVs are genetically diverse and are classified into six established genogroups (GI–GVI) based on VP1 sequences [10, 14]. Of the six genogroups, GI, GII, and GIV infect humans, and GII is the most common threat, causing 75–90% of all HNoV-related outbreaks [11, 12, 17, 18]. To date, nine capsid genotypes have been identified in GI, 22 in GII, and three genotypes of GII (GII.11, GII.18, and GII.19) have been uniquely detected in swine. Of the two genotypes of GIV identified to date, GIV.1 can infect humans [10].

HNoV is known to spread through the fecal-oral route, which can be subdivided into direct person-to-person contact (88%), food ingestion (10%), and drinking water intake (1.5%) [19]. Epidemiological studies show that HNoVs can survive for prolonged periods outside of the host [2]. To date, studies for HNoV detection in water have revealed that HNoVs are present in aquatic environments such as raw/treated sewage [20], rivers [20–26], groundwater [12, 18, 27, 28], ocean water [2, 24, 29, 30], and tap water [31]. In particular, contamination of the marine environment with viruses from the human community increases the potential for outbreaks via recreation and shellfish consumption [32].

The objective of this study was to investigate the distribution over time of the GI and GII genotypes of NoV in estuaries and inflow streams in four different geographical areas in South Korea. This study is the first nationwide study conducted in South Korea focusing on detection of HNoV contamination in coastal environments that are utilized as clam fisheries.

## Materials and Methods

### Ethics statement

Sample collection was approved by the Korean Food and Drug Administration (KFDA, Project No. 14162–973). This study did not require additional permissions because samples were not collected on private land or in protected areas. We confirm that this study did not involve endangered or protected species.

### Water sample collection and processing

**Fig 1:**
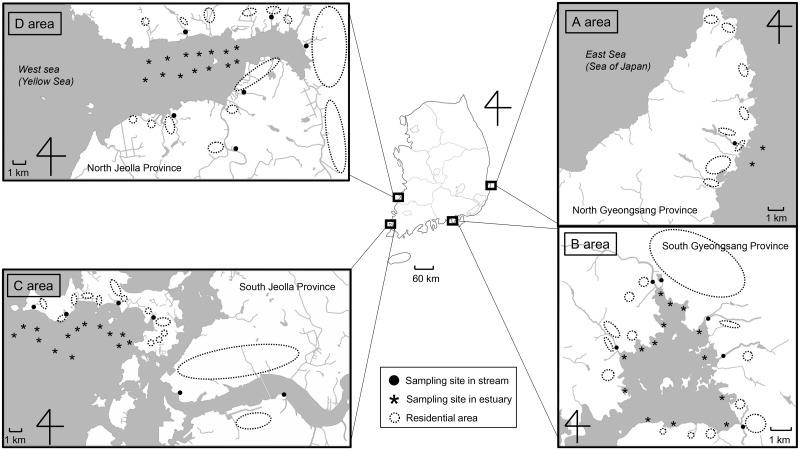
Locations of the sampling sites and residential areas of the study area in South Korea.

From March 2014 to February 2015, water samples were collected from four peri-urban coastal regions located in the eastern (area A), southern (area B), southwestern (area C), and western (area D) areas of South Korea (Fig 1). Each study area included one estuary and between one and six inflow streams (one stream in area A; six streams in each of areas B, C, and D). Domestic sewage from dwellings flows into each of the neighboring streams. The estuary in area A was sampled at two different sites, and each estuary in areas B, C, and D was sampled at 14 different sites. The estuary in area A includes a limited number of inflow streams and sampling sites owing to its geographical features. Each inflow stream was sampled at one site. Thus, samples were collected from a total of 63 sites in the four areas: 44 sites in four estuaries which have been used as clam fisheries and 19 sites in 19 inflow streams. The 63 sites were sampled eight times during the study period (Mar–Apr, May, Jul, Aug, Sep, Oct, Jan, and Feb). Thus, a total of 504 samples were collected.

According to the standard procedure [33, 34], less than 100 L of water was filtrated through NanoCeram cartridge filters (Argonide, Sanford, FL, USA). The filters were stored at 4°C and concentrated according to method 1615 within 72 h of sample collection [35]. Final concentrates were stored at -70°C until analysis. Additional water samples were collected using sterile polypropylene bottles for analysis of physicochemical characteristics and fecal indicator bacteria.

### RNA extraction and nucleic acid amplification

Viral RNA was extracted from the water concentrate using a QIAamp viral RNA mini kit (Qiagen, Hilden, Germany) according to the manufacturer’s protocol, in a final volume of 60 μL. The RNA was stored at -70°C until RT-PCR analysis.

The primer sets used to amplify the NoV GI and GII target regions are listed in Table 1 [36]. Region C [37] of NoV GI and GII was first amplified using a Verso 1-step RT-PCR ReddyMix kit (Thermo Fisher Scientific, Waltham, MA, USA) according to the manufacturer’s protocol, with minor modifications. Briefly, the RNA (5 μL) extracted from each water sample was mixed with 1-Step PCR ReddyMix (9.5 μL), the forward and reverse primers (40 μM each), and Verso Enzyme Mix (0.5 μL). Deionized sterile water was added up to a final reaction volume of 25 μL. The one-step RT-PCR conditions were as follows: reverse transcription at 45°C for 30 min, denaturation at 95°C for 5 min, and 35 cycles of amplification (94°C for 30 s, 55°C for 30 s, and 72°C for 90 s), plus a final extension at 72°C for 7 min. For the second semi-nested PCR amplification, *Top* DNA polymerase (Bioneer, Daejeon, South Korea) was used according to the manufacturer’s protocol, with minor modifications. Briefly, each RT-PCR product (2 μL) was mixed with 10× buffer (5 μL), 10 mM dNTP mix (4 μL), forward and reverse primers (50 μM each), and DNA polymerase (5 U). Deionized sterile water was added up to a final reaction volume of 50 μL. The semi-nested PCR conditions were as follows: initial denaturation at 94°C for 5 min and 25 cycles of amplification (94°C for 30 s, 55°C for 30 s, and 72°C for 90 s), plus a final extension at 72°C for 7 min.

**Table 1:** Primers used in this study.

Genogroup	Primer ID	Sequences (5′→3′)^c^	Position^d^	Polarity
I	GI-F1M ^a^	CTGCCCGAATTYGTAAATGATGAT	5341–5364	Sense
	GI-R1M ^a^^,^^b^	CCAACCCARCCATTRTACATYTG	5648–5670	Antisense
	GI-F2 ^b^	ATGATGATGGCGTCTAAGGACGC	5357–5379	Sense
II	GII-F1M ^a^	GGGAGGGCGATCGCAATCT	5048–5063	Sense
	GII-R1M^a^^,^^b^	CCRCCNGCATRICCRTTRTACAT	5366–5388	Antisense
	GII-F3M ^b^	TTGTGAATGAAGATGGCGTCGART	5078–5101	Sense

^a^ Primer sets for one-step RT-PCR

^b^ Primer sets for semi-nested PCR

^c^ “Y” = C/T; “R” = A/G; “N” = A/C/G/T

^d^ Primer positions of NoV GI are based on GenBank ID JX023285, and primer positions for NoV GII are based on GenBank ID JQ622197.

### Cloning and sequence analysis

Target amplicons were separated by electrophoresis and purified using a MiniBEST Agarose Gel DNA Extraction Kit Ver. 4.0 (Takara, Kusatsu, Japan) following the manufacturer’s protocol. The purified amplicons were cloned using both a Mighty TA-cloning Kit (Takara) and chemically competent DH5 α (Enzynomics, Daejeon, South Korea). Six colonies for each amplicon were chosen for seeding in liquid medium for culture. Cloned genes were purified using a HiYield^™^ Plasmid Mini Kit (RBC, Banqiao, Taiwan) and sequenced (Macrogen Seoul, South Korea) using a 3730xl DNA analyzer (Thermo Fisher Scientific).

### Measurement of physicochemical parameters and fecal indicator bacteria

The physicochemical parameters of temperature (°C), conductivity (uS/cm), pH, and turbidity (NTU) were measured for each sample using an Orion 4-star pH/conductivity meter (Thermo Fisher Scientific). Fecal coliform and *E*. *coli* were measured using the 5-tube most probable number (MPN) method [38]. Briefly, each diluted water sample was first inoculated into lauryl tryptose broth and incubated at 35°C for 48 h. Cultures that generated gas were transferred into both brilliant green bile broth (Oxoid, Hampshire, UK) and *E*. *coli* broth (BD, Franklin Lakes, NJ, USA) to test for fecal coliform and *E*. *coli*. After incubation, positive tubes were counted and assessed using MPN tables.

### Phylogenetic and statistical analysis

HNoV sequences from the water samples were aligned using Clustal W, version 1.81. Phylogenetic relationships among the sequences were determined using Molecular Evolutionary Genetics Analysis software (version 6.0). The Kimura 2-parameter model was used as the substitution method, and neighbor-joining was used to construct phylogenetic trees with 1000 bootstrap replicates. Statistical analysis was performed using the Statistical Package for the Social Sciences software (version 22.0.0). Independent samples *t*-tests were used to compare the mean value of each environmental factor in NoV-positive samples and the mean value of the same environmental factor in NoV-negative samples [39]. *P*-values < 0.05 were considered to indicate significance.

### Accession numbers for isolated sequences

The nucleotide sequences were deposited in the GenBank database with the following accession numbers: KT383850–KT384078.

## Results

### HNoV detection in estuaries and inflow streams

From March 2014 to February 2015, we collected a total of 504 water samples (352 estuary samples and 152 inflow stream samples) from a total of 63 sites in four coastal areas in South Korea (Fig 1). Each area for water sampling contained one estuary and its inflow streams. From estuaries, we collected 16 samples from area A and 112 samples from each of areas B-D. From inflow streams, we collected eight samples from area A and 48 samples from each of areas B-D. Using RT-PCR targeting NoV GI and GII, HNoVs were detected in 104 of the total 504 samples (7.9% of the estuarine water samples and 50.0% of the inflow stream water samples; Table 2). The rates of HNoV detection revealed regional differences within each of the two water types. Among the estuary samples, those from area B showed the highest rate of HNoV detection (11.6%), followed by area D (8.0%), area A (6.2%), and area C (4.5%). Among the inflow stream samples, those from area A showed the highest rate of HNoV detection (87.5%), followed by area B (64.6%), area D (47.9%), and area C (31.2%).

**Table 2:** NoV GI and GII detection rates in estuaries and their inflow streams in South Korea.

Area	The number of HNoV-positive samples; n(percent positive)								
	Estuary			Stream			Estuary + Stream		
	GI	GII	Total^a^	GI	GII	Total^a^	GI	GII	Total^a^
A, B, C, D	7(1.9%)	23(6.5%)	28(7.9%)	60(39.4%)	61(40.1%)	76(50.0%)	67(13.3%)	84(16.6%)	104(20.6%)
A	1(6.2%)	1(6.2%)	1(6.2%)	5(62.5%)	6(75.0%)	7(87.5%)	6(25.0%)	7(29.2%)	8(33.3%)
B	5(4.5%)	9(8.0%)	13(11.6%)	27(56.2%)	24(50.0%)	31(64.6%)	32(20.0%)	33(20.6%)	44(27.5%)
C	0(0.0%)	5(4.5%)	5(4.5%)	11(22.9%)	14(29.2%)	15(31.2%)	11(6.9%)	19(11.9%)	20(12.5%)
D	1(0.9%)	8(7.1%)	9(8.0%)	17(35.4%)	17(35.4%)	23(47.9%)	18(11.2%)	25(15.6%)	32(20.0%)

^a^ Norovirus-positive samples of both genotypes combined

**Fig 2:**
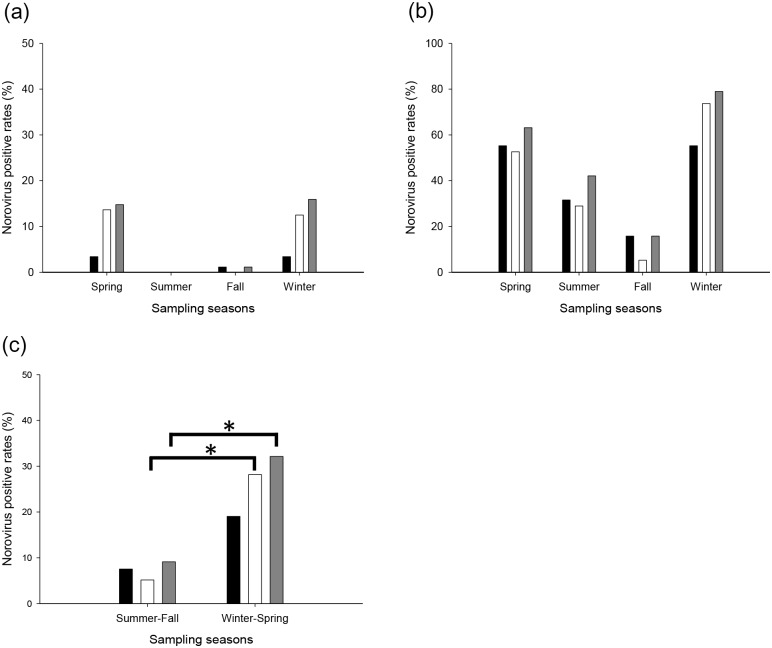
Seasonal detection rates of human norovirus (HNoV) in water samples. HNoV detection rates in spring (Mar to May), summer (July to Aug), fall (Sep to Oct), and winter (Jan to Feb) according to water type: (a) estuary and (b) inflow stream. The colors of the bars indicate genogroups: GI (black), GII (white), or HNoV (combination of the two genogroups, grey). Detection rates independent of water type in winter/spring and summer/fall are shown in panel (c). The colors of the bars indicate genogroups: GI (black), GII (white), or HNoV (combination of the two genogroups, grey). Asterisks (*) indicate significant differences (*P* < 0.05).

Both estuary and stream water samples collected in spring (Mar to May) and winter (Jan to Feb) showed higher rates of HNoV detection (Fig 2a and 2b). Synthetically, detection rates of GII (28.2%) and the combination of two genogroups (GI and GII, 32.1%) in winter/spring (Jan to Feb and Mar to May) samples were significantly (*P* < 0.05) higher than in summer/fall (July to Aug and Sep to Oct) samples (5.2% for GII, and 9.1% for the combination of the two genogroups) (Fig 2c). Although it was not significant (*P* > 0.05), the detection rate of GI (19.0%) in winter/spring samples was higher than in summer/fall (7.5%).

### Correlation between environmental factors and HNoV occurrence in water samples

The measured values of both physicochemical factors and fecal indicator bacteria are listed according to season and water type (Table 3). Water temperature was lowest in winter, followed by spring, fall, and summer in both water types. The MPN values of total coliform and *E*. *coli* were highest in summer and lowest in winter in both water types. A *t*-test to detect associations between environmental factors and HNoV occurrences revealed that water temperature was significantly correlated with HNoV occurrence in three of the four areas, as well as overall (*P* < 0.05; Table 4). In estuaries, conductivity was correlated with HNoV occurrence overall; but significant correlations (*P* < 0.05) were observed in only two of the four individual areas.

**Table 3:** Mean of physicochemical and fecal indicator bacteria values.

Water type	Sampling season	Water temperature [°C]	pH	Turbidity [NTU] ^b^	Conductivity [uS/cm]	Total coliform [log MPN^c^/100 ml]	*Escherichia coli* [log MPN/100 ml]
Estuary	Spring	15.5^a^	7.8	6.17	53798	0.6	0.5
	Summer	24.3	7.8	7.19	46273	0.9	0.7
	Fall	22.0	7.7	5.72	46289	0.5	0.4
	Winter	6.0	8.1	10.83	38542	0.3	0.2
Stream	Spring	18.3	7.3	11.6	6454	2.7	2.3
	Summer	24.1	7.5	18.7	3659	3.5	2.7
	Fall	22.0	7.6	9.2	7894	3.3	2.3
	Winter	6.6	7.4	8.8	7228	2.6	1.6

^a^ Mean

^b^ Nephelometric turbidity unit

^c^ Most probable number

**Table 4:** Correlation between HNoV occurrence and environmental factors, determined using *t*-tests.

	Area A		Area B		Area C		Area D		Overall^b^	
Environmental factor	Estuary	Stream	Estuary	Stream	Estuary	Stream	Estuary	Stream	Estuary	Stream
Water temperature	0.486	0.238	0.013*^a^	0.001*	0.023*	0.009*	0.000*	0.000*	0.000*	0.000*
pH	0.232	0.389	0.078	0.106	0.640	0.043*	0.692	0.000*	0.132	0.669
Turbidity	0.505	0.060	0.076	0.756	0.211	0.880	0.117	0.852	0.666	0.321
Conductivity	0.840	0.265	0.000*	0.024*	0.522	0.721	0.035*	0.816	0.001*	0.363
Total coliform	0.738	0.433	0.356	0.658	0.579	0.534	0.609	0.303	0.575	0.538
*Escherichia coli*	0.733	0.438	0.555	0.434	0.649	0.337	0.745	0.226	0.491	0.245

^a^ *P*-values < 0.05 are indicated by asterisks (*). *P* < 0.05 signifies that mean values of a particular environmental factor differ significantly between NoV-positive and NoV-negative samples.

^b^ Total area

### Genotypic diversity and prevalence of NoV GI and GII in environmental water samples

**Fig 3:**
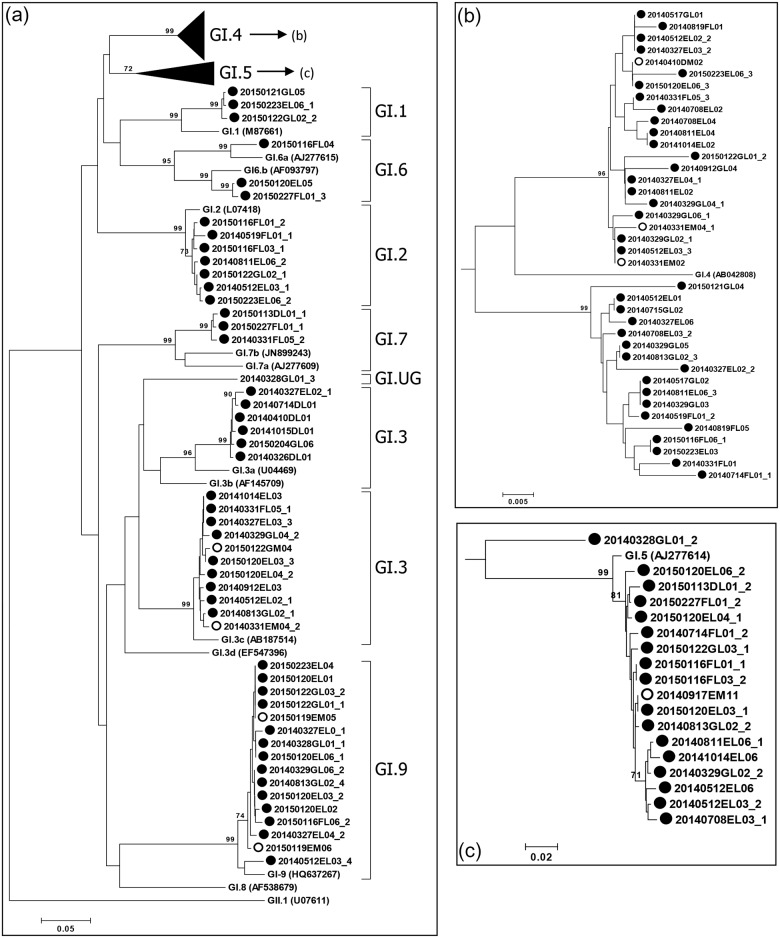
Phylogenetic analysis of human norovirus (HNoV) genogroup I (GI) nucleotide sequences isolated in this study. Phylogenetic tree based on partial capsid gene sequences (a). Clusters of GI.4 (b) and GI.5 (c) sequences are depicted separately. GII.1 (GenBank ID; U07661) was used as the outgroup reference sequence. Norovirus sequences isolated in this study are marked with open circles (○, estuary) and filled circles (●, inflow stream). Suffixes (_1, _2 and so forth) indicate different sequences originating from a single sample. Bootstrap values ≥70 are shown at each branch, indicating groupings with significant support. The scale bar represents nucleotide substitutions per site.

**Fig 4:**
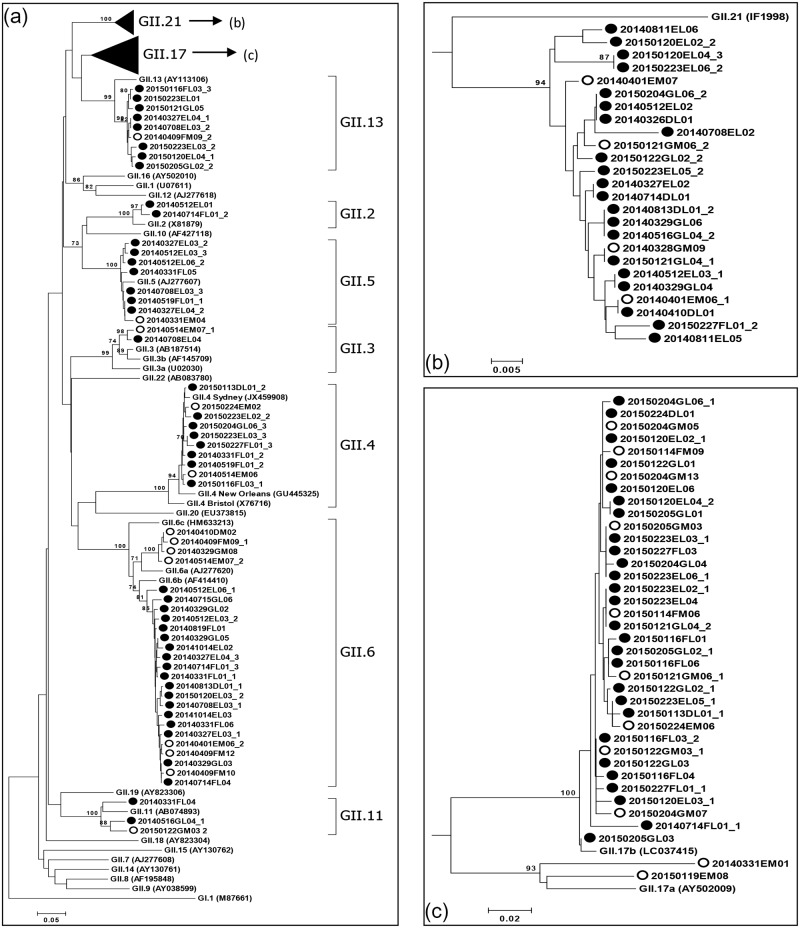
Phylogenetic analysis of human norovirus (HNoV) genogroup II (GII) nucleotide sequences isolated in this study. Phylogenetic tree based on partial capsid gene sequences (a). Clusters of GII.21 (b) and GII.17 (c) sequences are depicted separately. GI.1 (GenBank ID; M87661) was used as the outgroup reference sequence. Norovirus sequences isolated in this study are marked with open circles (○, estuary) and filled circles (●, inflow stream). Suffixes (_1, _2 and so forth) indicate different sequences originating from a single sample. Bootstrap values ≥70 are shown at each branch, indicating groupings with significant support. The scale bar represents nucleotide substitutions per site.

**Fig 5:**
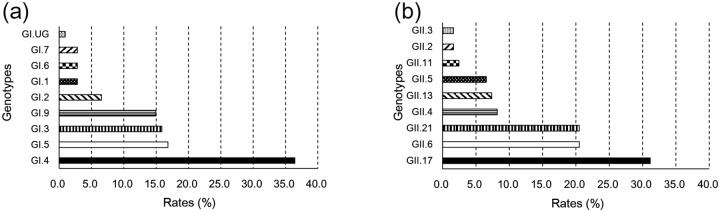
Prevalence of human norovirus (HNoV) genotypes in water samples. Relative prevalence of genotypes of GI sequences (a) and all GII sequences (b). Each pattern of horizontal bars indicates a distinct genotype.

**Fig 6:**
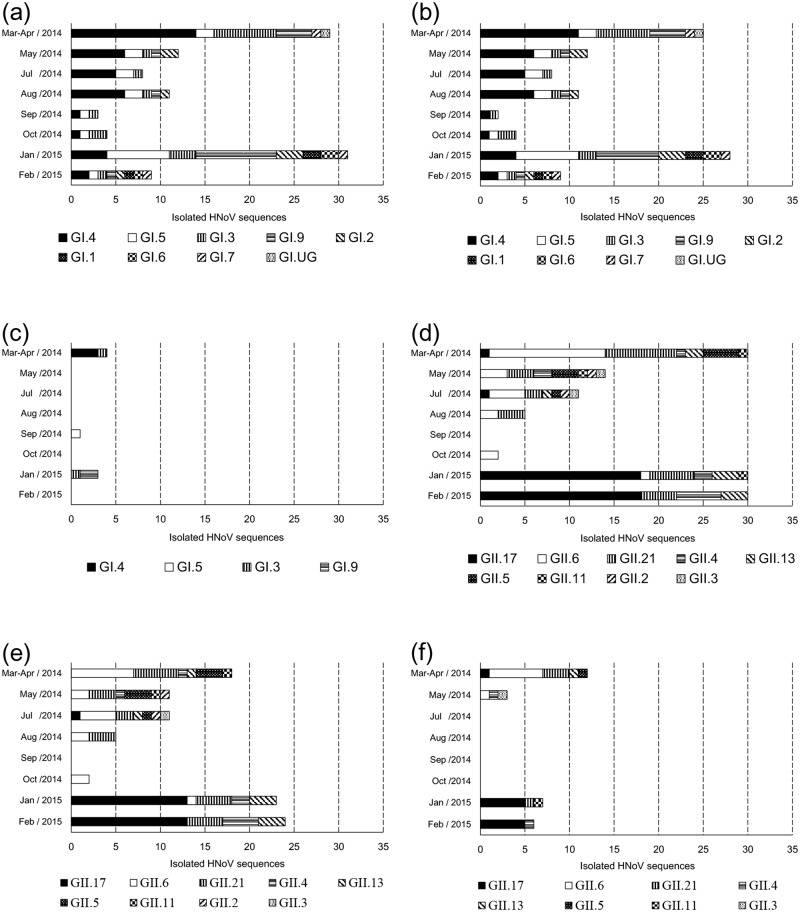
Comparison of the number of human norovirus (HNoV) sequences isolated from each water type by sampling month. The number of HNoV sequences isolated is shown in the horizontal stacked bar charts. Charts show genogroups (a–c = GI; d–f = GII) and water types (a and d = both stream and estuary; b and e = stream; c and f = estuary). In each panel, the HNoV sequences are sorted by sampling month (vertical axis), and the internal patterning of the horizontal bars (total HNoV sequences in a sampling month) indicates the number of sequences of each genotype.

**Fig 7:**
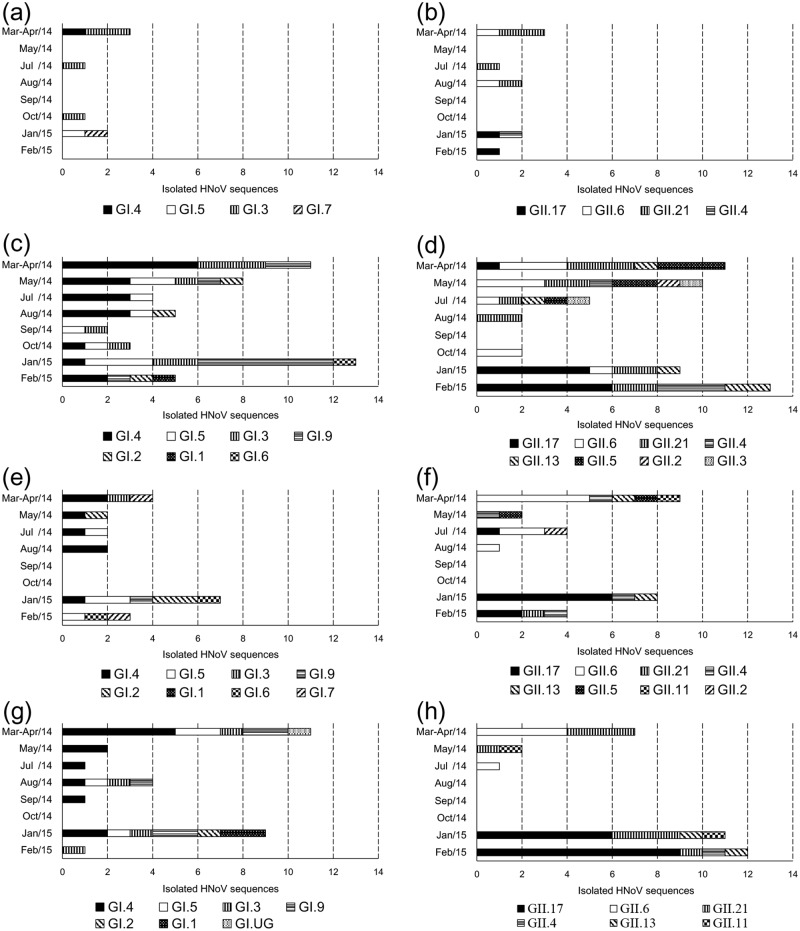
Comparison of the number of human norovirus (HNoV) sequences isolated in each of the four areas by sampling month. The number of HNoV sequences isolated is shown in the horizontal stacked bar charts. Charts show genogroups (a, c, e, and g = GI; b, d, f, and h = GII) and study areas (a and b = area A; c and d = area B; e and f = area C; g and h = area D). In each panel, the HNoV sequences are sorted by sampling month (vertical axis), and the internal patterning of the horizontal bars (total HNoV sequences in a sampling month) indicates the number of sequences of each genotype.

We conducted a phylogenetic analysis of region C (5′-end of ORF2, 0.3 kb) to analyze the relationships between the 229 HNoV sequences (107 GI sequences and 122 GII sequences) and genotypic reference sequences, using the neighbor-joining method. Except for one unclassified genotype (GI.UG), all GI sequences were clustered into eight genotypes: GI.1, GI.2, GI.3, GI.4, GI.5, GI.6, GI.7, and GI.9 (Fig 3); the GII sequences were clustered into nine genotypes: GII.2, GII.3, GII.4, GII.5, GII.6, GII.11, GII.13, GII.17, and GII.21 (Fig 4). The three major GI genotypes were GI.4 (39 sequences; 36.4%), GI.5 (18 sequences; 16.8%), and GI.3 (17 sequences; 15.9%). The three major GII genotypes were GII.17 (38 sequences; 31.1%), GII.6 (25 sequences; 20.5%), and GII.21 (25 sequences; 20.5%) (Fig 5). Although local differences were detected, the majority of the GI.4, GI.3, and GII.6 sequences were detected in the first half (Mar-Aug; spring/summer) of the sampling period and the majority of the GI.5, GI.9, GII.4, and GII.17 sequences were detected in the latter half (Sep-Feb; fall/winter) of the sampling period (Figs 6 and 7).

## Discussion

Human activity influences the presence and prevalence of human norovirus in the aquatic environment [40], and outbreaks caused by waterborne viruses have caused serious health and socio-economic impacts [41]. Although the presence of HNoVs in clams has been widely reported [9, 42–46], HNoV monitoring in coastal environments is relatively limited [2, 17, 24]. In this study, we investigated the prevalence, seasonal dependence, and genotypic diversity of both NoV GI and GII in four different estuaries (clam fisheries) and their inflow streams, which were in the vicinity of human dwellings in South Korea.

In this study, HNoVs were detected in 7.9% of estuary water samples and 50.0% of inflow stream water samples. The difference in detection rates between estuaries and streams might be due to the greater dispersion/dilution effects and lower stability of viruses in the marine environment [24]. The detection rate did not differ significantly between the two genogroups in streams (*P* > 0.05; Table 2), concordant with the results of previous studies of NoV in rivers [20, 25]. GII was slightly more prevalent in estuaries than GI, although the difference was not significant (*P* > 0.05), suggesting the possible presence of a genotype-specific PCR inhibitor or water type—dependent GI capsid instability. Nevertheless, these environmental observations did not coincide with the overwhelming dominance of GII in hospitalized cases [47–50], which might suggest that large-scale asymptomatic infection with NoV GI occurs in South Korea.

In this study, 17 different genotypes and one undefined genotype were isolated from the water samples: GI.1, GI.2, GI.3, GI.4, GI.5, GI.6, GI.7, GI.9, GI.UG, GII.2, GII.3, GII.4, GII.5, GII.6, GII.11, GII.13, GII.17, and GII.21 (Figs 3 and 4). The observed genotypic diversity is comparable to that reported from river samples collected in South Korea (15 genot**y**pes) [25], Japan (16 genotypes) [21], South Africa (16 genotypes) [20], and from wastewater in Singapore (19 genotypes) [51]. Although local/time differences in genotype occurrence were observed, the detection frequency of GI.4, GI.3, and GII.6 peaked in the first half of the sampling period, while the detection frequency of GI.5, GI.9, GII.4, and GII.17 peaked in the latter half of the sampling period (Figs 6 and 7). Establishment of temporal herd immunity may have influenced the transition between major genotypes. The concurrent presence of these different genotypes suggests that various genotypes cause asymptomatic infections, and also suggests the possibility that non-GII.4 noroviruses will trigger major outbreaks when the GII.4-host relationship changes.

GII.4 has been the dominant genotype in HNoV-associated outbreaks worldwide [45, 50, 52–55]. Recent reports, however, showed that novel GII.17 NoVs, not GII.4 pandemic variants, have been the major causative agent of recent outbreaks in China and Japan [56–59]. Interestingly, GII.4 in this study was not the dominant GII genotype; GII.6 was dominant in 2014 and GII.17 was dominant in 2015 (Figs 6 and 7). The GII.17 sequences identified in this study were clustered with the previously reported novel GII.17 variants in the phylogenetic analysis. The novel GII.17 genotype detected in this study was first detected in July 2014 (20140714FL01_1, reference sequence: LC037415, Fig 4c), and the GII.17 sequences were detected at highest frequency in the following winter. Thus, the GII.17 NoV that is dominant in East Asia could pose an additional major threat on other continents, and we recommend that current studies, including vaccine development, concentrating on GII.4 pandemic variants should be extended to other genotypes. Because current *in vivo*/*in vitro* infection systems for study of HNoV are limited, the underlying cause of the disproportionate detection of one or two genotypes in most clinical samples cannot be fully investigated at present.

It is commonly accepted that HNoV-related diseases tend to occur in winter [24], and seasonal HNoV occurrence was higher in spring/winter than in summer/fall (Fig 2c). A previous study showed that environmental NoVs were more frequently detected in winter/spring [21]. The correlation between water temperature and HNoV occurrence support the seasonality of HNoV (Table 4). Although the correlation between water temperature and HNoV detection in area A was not significant (*P* > 0.05), this may have been due to the relatively small sample size. Because winter is the main season for harvest of clams in South Korea, the highest viral prevalence observed in winter estuary samples may increase the norovirus load in clams. In fact, the major genotypes detected in inflow streams were also frequently detected in all four estuaries (S1 and S2 Figs), suggesting that streams could affect norovirus inflow into the marine environment; thus, continuous surveillance efforts and construction of sanitation facilities at the lower streams that affect clam fisheries will help to reduce seafood-related HNoV outbreaks.

Thus far, few reports have investigated the prevalence of HNoVs in coastal environments, and to the best of our knowledge, this is the first comprehensive report on HNoV prevalence in estuaries and their inflow streams in South Korea. Environmental monitoring for HNoV will provide data that will be helpful for resolution of seasonal epidemics in future. Although this study preferentially focused on correlations between HNoV contamination of estuaries and their inflow streams, a recent report demonstrated that the novel GII.17 variant was second in predominance among all NoV-positive children diagnosed with acute gastroenteritis from 2013–2015 in South Korea, indicating the importance of monitoring the aquatic environment [60].

This study provides important basic data on 1) the inflow of HNoV into estuaries harboring clam fisheries and 2) the temporal distribution/transition of HNoV in the aquatic environment, which can provide important data for identifying novel genotypes that may cause epidemics in the near future.

## Supporting Information

S1 Fig Comparison of the number of isolated NoV GI sequences on sampling months. The numbers of isolated NoV GI sequences are described as horizontal stacked bar charts. Charts are illustrated by combination between study areas (a and b = area A; c and d = area B; e and f = area C; g and h = area D) and water types (a, c, e, and g = stream; b, d, f, and h = estuary). In each panel, NoV GI sequences are sorted by those sampling months (vertical axis), and each horizontal bar (total NoV GI sequences in a sampling month) is consist of internal patterns (the number of sequences for identified genotypes).
(TIF)

S2 Fig Comparison of the number of isolated NoV GII sequences on sampling months. The numbers of isolated NoV GII sequences are described as horizontal stacked bar charts. Charts are illustrated by combination between study areas (a and b = area A; c and d = area B; e and f = area C; g and h = area D) and water types (a, c, e, and g = stream; b, d, f, and h = estuary). In each panel, NoV GII sequences are sorted by sampling months (vertical axis), and each horizontal bar (total NoV GII sequences in a sampling month) is consist of internal patterns (the number of sequences for identified genotypes).
(TIF)

S1 Table The complete data set for this study. (XLSX)

## References

[1] Ahmed SM, Hall AJ, Robinson AE, Verhoef L, Premkumar P, Parashar UD, et al Global prevalence of norovirus in cases of gastroenteritis: a systematic review and meta-analysis. Lancet Infect Dis. 2014;14(8):725–30. 10.1016/S1473-3099(14)70767-4 . 24981041 PMC8006533

[2] Yang N, Qi H, Wong MM, Wu RS, Kong RY. Prevalence and diversity of norovirus genogroups I and II in Hong Kong marine waters and detection by real-time PCR. Mar Pollut Bull. 2012;64(1):164–8. 10.1016/j.marpolbul.2011.10.037 . 22119412

[3] Yu Y, Yan S, Li B, Pan Y, Wang Y. Genetic diversity and distribution of human norovirus in China (1999–2011). Biomed Res Int. 2014;2014:196169 10.1155/2014/196169 24672783 PMC3918700

[4] Haramoto E, Katayama H, Utagawa E, Ohgaki S. Recovery of human norovirus from water by virus concentration methods. J Virol Methods. 2009;160(1–2):206–9. 10.1016/j.jviromet.2009.05.002 . 19447140

[5] Ramani S, Atmar RL, Estes MK. Epidemiology of human noroviruses and updates on vaccine development. Curr Opin Gastroenterol. 2014;30(1):25–33. 10.1097/MOG.0000000000000022 24232370 PMC3955997

[6] Kittigul L, Pombubpa K, Taweekate Y, Yeephoo T, Khamrin P, Ushijima H. Molecular characterization of rotaviruses, noroviruses, sapovirus, and adenoviruses in patients with acute gastroenteritis in Thailand. J Med Virol. 2009;81(2):345–53. 10.1002/jmv.21380 . 19107961

[7] Lopman BA, Reacher MH, Vipond IB, Sarangi J, Brown DW. Clinical manifestation of norovirus gastroenteritis in health care settings. Clin Infect Dis. 2004;39(3):318–24. 10.1086/421948 . 15306997

[8] Phan TG, Kuroiwa T, Kaneshi K, Ueda Y, Nakaya S, Nishimura S, et al Changing distribution of norovirus genotypes and genetic analysis of recombinant GIIb among infants and children with diarrhea in Japan. J Med Virol. 2006;78(7):971–8. 10.1002/jmv.20649 . 16721850

[9] Gallimore CI, Cheesbrough JS, Lamden K, Bingham C, Gray JJ. Multiple norovirus genotypes characterised from an oyster-associated outbreak of gastroenteritis. Int J Food Microbiol. 2005;103(3):323–30. 10.1016/j.ijfoodmicro.2005.02.003 . 15967530

[10] Vinje J. Advances in laboratory methods for detection and typing of norovirus. J Clin Microbiol. 2015;53(2):373–81. 10.1128/JCM.01535-14 24989606 PMC4298492

[11] Zheng DP, Ando T, Fankhauser RL, Beard RS, Glass RI, Monroe SS. Norovirus classification and proposed strain nomenclature. Virology. 2006;346(2):312–23. 10.1016/j.virol.2005.11.015 . 16343580

[12] Jung JH, Yoo CH, Koo ES, Kim HM, Na Y, Jheong WH, et al Occurrence of norovirus and other enteric viruses in untreated groundwaters of Korea. J Water Health. 2011;9(3):544–55. 10.2166/wh.2011.142 . 21976201

[13] Katayama K, Shirato-Horikoshi H, Kojima S, Kageyama T, Oka T, Hoshino F, et al Phylogenetic analysis of the complete genome of 18 Norwalk-like viruses. Virology. 2002;299(2):225–39. . 12202225 10.1006/viro.2002.1568

[14] Green KY. Caliciviridae: the noroviruses In: Knipe DM, Howley PM, Cohen JI, Griffin DE, Lamb RA, Martin MA, et al, editors. Fields virology, 6th ed Lippincott Williams & Wilkins, Philadelphia, PA 2013 p. 508–609.

[15] Hardy ME. Norovirus protein structure and function. FEMS Microbiol Lett. 2005;253(1):1–8. 10.1016/j.femsle.2005.08.031 . 16168575

[16] Bok K, Green KY. Norovirus gastroenteritis in immunocompromised patients. N Engl J Med. 2012;367(22):2126–32. 10.1056/NEJMra1207742 . 23190223 PMC4944753

[17] La Rosa G, Fontana S, Di Grazia A, Iaconelli M, Pourshaban M, Muscillo M. Molecular identification and genetic analysis of Norovirus genogroups I and II in water environments: comparative analysis of different reverse transcription-PCR assays. Appl Environ Microbiol. 2007;73(13):4152–61. 10.1128/AEM.00222-07 17483265 PMC1932759

[18] Lee SG, Jheong WH, Suh CI, Kim SH, Lee JB, Jeong YS, et al Nationwide groundwater surveillance of noroviruses in South Korea, 2008. Appl Environ Microbiol. 2011;77(4):1466–74. 10.1128/AEM.01996-10 21183642 PMC3067240

[19] Kroneman A, Verhoef L, Harris J, Vennema H, Duizer E, van Duynhoven Y, et al Analysis of integrated virological and epidemiological reports of norovirus outbreaks collected within the Foodborne Viruses in Europe network from 1 July 2001 to 30 June 2006. J Clin Microbiol. 2008;46(9):2959–65. 10.1128/JCM.00499-08 18650354 PMC2546741

[20] Mans J, Netshikweta R, Magwalivha M, Van Zyl WB, Taylor MB. Diverse norovirus genotypes identified in sewage-polluted river water in South Africa. Epidemiol Infect. 2013;141(2):303–13. 10.1017/S0950268812000490 . 22436724 PMC9152073

[21] Kitajima M, Oka T, Haramoto E, Takeda N, Katayama K, Katayama H. Seasonal distribution and genetic diversity of genogroups I, II, and IV noroviruses in the Tamagawa River, Japan. Environ Sci Technol. 2010;44(18):7116–22. 10.1021/es100346a . 20715862

[22] Gentry J, Vinje J, Guadagnoli D, Lipp EK. Norovirus distribution within an estuarine environment. Appl Environ Microbiol. 2009;75(17):5474–80. 10.1128/AEM.00111-09 19581478 PMC2737928

[23] Kitajima M, Haramoto E, Phanuwan C, Katayama H, Ohgaki S. Detection of genogroup IV norovirus in wastewater and river water in Japan. Lett Appl Microbiol. 2009;49(5):655–8. 10.1111/j.1472-765X.2009.02718.x . 19780954

[24] Wyn-Jones AP, Carducci A, Cook N, D'Agostino M, Divizia M, Fleischer J, et al Surveillance of adenoviruses and noroviruses in European recreational waters. Water Res. 2011;45(3):1025–38. 10.1016/j.watres.2010.10.015 . 21093010 PMC7112131

[25] Lee C, Kim SJ. The genetic diversity of human noroviruses detected in river water in Korea. Water Res. 2008;42(17):4477–84. 10.1016/j.watres.2008.08.003 . 18778846

[26] Perez-Sautu U, Sano D, Guix S, Kasimir G, Pinto RM, Bosch A. Human norovirus occurrence and diversity in the Llobregat river catchment, Spain. Environ Microbiol. 2012;14(2):494–502. 10.1111/j.1462-2920.2011.02642.x . 22118046

[27] Lambertini E, Spencer SK, Bertz PD, Loge FJ, Kieke BA, Borchardt MA. Concentration of enteroviruses, adenoviruses, and noroviruses from drinking water by use of glass wool filters. Applied and Environmental Microbiology. 2008;74(10):2990–6. 10.1128/AEM.02246-07 18359827 PMC2394941

[28] Joung HK, Han SH, Park SJ, Jheong WH, Ahn TS, Lee JB, et al Nationwide surveillance for pathogenic microorganisms in groundwater near carcass burials constructed in South Korea in 2010. Int J Environ Res Public Health. 2013;10(12):7126–43. 10.3390/ijerph10127126 24351737 PMC3881157

[29] Tong H-I, Connell C, Boehm AB, Lu Y. Effective detection of human noroviruses in Hawaiian waters using enhanced RT-PCR methods. Water research. 2011;45(18):5837–48. 10.1016/j.watres.2011.08.030 21945082

[30] Katayama H, Shimasaki A, Ohgaki S. Development of a virus concentration method and its application to detection of enterovirus and norwalk virus from coastal seawater. Appl Environ Microbiol. 2002;68(3):1033–9. 11872447 10.1128/AEM.68.3.1033-1039.2002 PMC123733

[31] Haramoto E, Katayama H, Ohgaki S. Detection of noroviruses in tap water in Japan by means of a new method for concentrating enteric viruses in large volumes of freshwater. Appl Environ Microbiol. 2004;70(4):2154–60. 15066808 10.1128/AEM.70.4.2154-2160.2004 PMC383138

[32] Gibbons CD, Rodriguez RA, Tallon L, Sobsey MD. Evaluation of positively charged alumina nanofibre cartridge filters for the primary concentration of noroviruses, adenoviruses and male-specific coliphages from seawater. J Appl Microbiol. 2010;109(2):635–41. 10.1111/j.1365-2672.2010.04691.x . 20202019

[33] Fout G, Schaefer F, Messer J, Dahling D, Stetler R. ICR microbial laboratory manual: US Environmental Protection Agency. National Exposure Research Laboratory: Washington, DC 1996.

[34] Parshionikar SU, Willian-True S, Fout GS, Robbins DE, Seys SA, Cassady JD, et al Waterborne outbreak of gastroenteritis associated with a norovirus. Appl Environ Microbiol. 2003;69(9):5263–8. 12957912 10.1128/AEM.69.9.5263-5268.2003 PMC194931

[35] Fout GS, Cashdollar JL, Varughese EA, Parshionikar SU, Grimm AC. EPA Method 1615. Measurement of Enterovirus and Norovirus Occurrence in Water by Culture and RT-qPCR. I. Collection of Virus Samples. J Vis Exp. 2015;(97). 10.3791/52067 25867928 PMC4401389

[36] Koo ES, Yoo CH, Na Y, Park SY, Lyoo HR, Jeong YS. Reliability of non-culturable virus monitoring by PCR-based detection methods in environmental waters containing various concentrations of target RNA. J Microbiol. 2012;50(5):726–34. 10.1007/s12275-012-2279-y . 23124739

[37] Vinjé J, Hamidjaja RA, Sobsey MD. Development and application of a capsid VP1 (region D) based reverse transcription PCR assay for genotyping of genogroup I and II noroviruses. J Virol Methods. 2004;116(2):109–17. . 14738976 10.1016/j.jviromet.2003.11.001

[38] American Public Health Association A. Recommended procedures for the examination of sea water and shellfish. APHA; 1970.

[39] Jovanović Galović A, Bijelović S, Milošević V, Hrnjaković Cvjetkovic I, Popović M, Kovačević G, et al Testing for viral material in water of public bathing areas of the Danube during summer, Vojvodina, Serbia, 2014. Euro Surveill. 2016;21(15). 10.2807/1560-7917.ES.2016.21.15.30196 . 27105473

[40] Aw TG, Gin KY, Ean Oon LL, Chen EX, Woo CH. Prevalence and genotypes of human noroviruses in tropical urban surface waters and clinical samples in Singapore. Appl Environ Microbiol. 2009;75(15):4984–92. 10.1128/AEM.00489-09 19525276 PMC2725494

[41] Grabow WO. Overview of health-related water virology. Human viruses in water. 2007;17:1–25. 10.1016/S0168-7069(07)17001-4 PMC7132496 32287590

[42] Nishida T, Kimura H, Saitoh M, Shinohara M, Kato M, Fukuda S, et al Detection, quantitation, and phylogenetic analysis of noroviruses in Japanese oysters. Appl Environ Microbiol. 2003;69(10):5782–6. 14532025 10.1128/AEM.69.10.5782-5786.2003 PMC201174

[43] Bigoraj E, Kwit E, Chrobocinska M, Rzezutka A. Occurrence of norovirus and hepatitis A virus in wild mussels collected from the Baltic Sea. Food Environ Virol. 2014;6(3):207–12. 10.1007/s12560-014-9153-5 . 24906970

[44] Nishida T, Nishio O, Kato M, Chuma T, Kato H, Iwata H, et al Genotyping and quantitation of noroviruses in oysters from two distinct sea areas in Japan. Microbiol Immunol. 2007;51(2):177–84. . 17310085 10.1111/j.1348-0421.2007.tb03899.x

[45] Seo DJ, Lee MH, Son NR, Seo S, Lee KB, Wang X, et al Seasonal and regional prevalence of norovirus, hepatitis A virus, hepatitis E virus, and rotavirus in shellfish harvested from South Korea. Food Control. 2014;41:178–84.

[46] Park J, Jeong H, Lee J, Lee S, Choi Y, Choi S, et al First norovirus outbreaks associated with consumption of green seaweed (Enteromorpha spp.) in South Korea. Epidemiology and infection. 2015;143(03):515–21. 10.1017/S0950268814001332 24866366 PMC9507068

[47] Won YJ, Park JW, Han SH, Cho HG, Kang LH, Lee SG, et al Full-genomic analysis of a human norovirus recombinant GII.12/13 novel strain isolated from South Korea. PLoS One. 2013;8(12):e85063 10.1371/journal.pone.0085063 24391985 PMC3877344

[48] Ham H, Oh S, Seung H, Jo S. Molecular Characteristics of Noroviruses Genogroup I and Genogroup II Detected in Patients With Acute Gastroenteritis. Annals of laboratory medicine. 2015;35(2):242–5. 10.3343/alm.2015.35.2.242 25729728 PMC4330176

[49] Cho HG, Lee SG, Kim JE, Yu KS, Lee DY, Park PH, et al Molecular epidemiology of norovirus GII.4 variants in children under 5 years with sporadic acute gastroenteritis in South Korea during 2006–2013. J Clin Virol. 2014;61(3):340–4. 10.1016/j.jcv.2014.08.018 . 25223918

[50] Jeong SY, Lee DY. Molecular Epidemiology of Norovirus-related Outbreaks in Korea, 2012–2013. Korea Centers for Disease Control and Prevention. 2014.

[51] Aw TG, Gin KY. Environmental surveillance and molecular characterization of human enteric viruses in tropical urban wastewaters. J Appl Microbiol. 2010;109(2):716–30. 10.1111/j.1365-2672.2010.04701.x . 20233263

[52] Maunula L, Miettinen IT, Von Bonsdorff C-H. Norovirus outbreaks from drinking water. Emerg Infect Dis. 2005;11(11):1716–21. 10.3201/eid1111.050487 16318723 PMC3367355

[53] Hoa-Tran TN, Nakagomi T, Sano D, Sherchand JB, Pandey BD, Cunliffe NA, et al Molecular epidemiology of noroviruses detected in Nepalese children with acute diarrhea between 2005 and 2011: increase and predominance of minor genotype GII.13. Infect Genet Evol. 2015;30:27–36. 10.1016/j.meegid.2014.12.003 . 25497351

[54] Fernandez MD, Torres C, Poma HR, Riviello-Lopez G, Martinez LC, Cisterna DM, et al Environmental surveillance of norovirus in Argentina revealed distinct viral diversity patterns, seasonality and spatio-temporal diffusion processes. Sci Total Environ. 2012;437:262–9. 10.1016/j.scitotenv.2012.08.033 . 22944218

[55] Verhoef L, Hewitt J, Barclay L, Ahmed SM, Lake R, Hall AJ, et al Norovirus genotype profiles associated with foodborne transmission, 1999–2012. Emerg Infect Dis. 2015;21(4):592–9. 10.3201/eid2104.141073 25811368 PMC4378480

[56] Kiulia N, Mans J, Mwenda J, Taylor M. Norovirus GII. 17 Predominates in Selected Surface Water Sources in Kenya. Food and environmental virology. 2014;6(4):221–31. 10.1007/s12560-014-9160-6 25059212

[57] Matsushima Y, Ishikawa M, Shimizu T, Komane A, Kasuo S, Shinohara M, et al Genetic analyses of GII.17 norovirus strains in diarrheal disease outbreaks from December 2014 to March 2015 in Japan reveal a novel polymerase sequence and amino acid substitutions in the capsid region. Euro Surveill. 2015;20(26). . 26159307 10.2807/1560-7917.es2015.20.26.21173

[58] Fu J, Ai J, Jin M, Jiang C, Zhang J, Shi C, et al Emergence of a new GII.17 norovirus variant in patients with acute gastroenteritis in Jiangsu, China, September 2014 to March 2015. Euro Surveill. 2015;20(24). . 26111236 10.2807/1560-7917.es2015.20.24.21157

[59] Lu J, Sun L, Fang L, Yang F, Mo Y, Lao J, et al Gastroenteritis Outbreaks Caused by Norovirus GII.17, Guangdong Province, China, 2014–2015. Emerg Infect Dis. 2015;21(7):1240–2. 10.3201/eid2107.150226 26080037 PMC4480401

[60] Dang Thanh H, Than VT, Nguyen TH, Lim I, Kim W. Emergence of Norovirus GII.17 Variants among Children with Acute Gastroenteritis in South Korea. PLoS One. 2016;11(5):e0154284 10.1371/journal.pone.0154284 27148739 PMC4858242

