# Supplementary figures and images for: Distribution of Human Norovirus in the Coastal Waters of South Korea
Source: PLoS One. 2016 Sep 28;11(9):e0163800. doi: 10.1371/journal.pone.0163800 (PMC5040428; doi:10.1371/journal.pone.0163800)

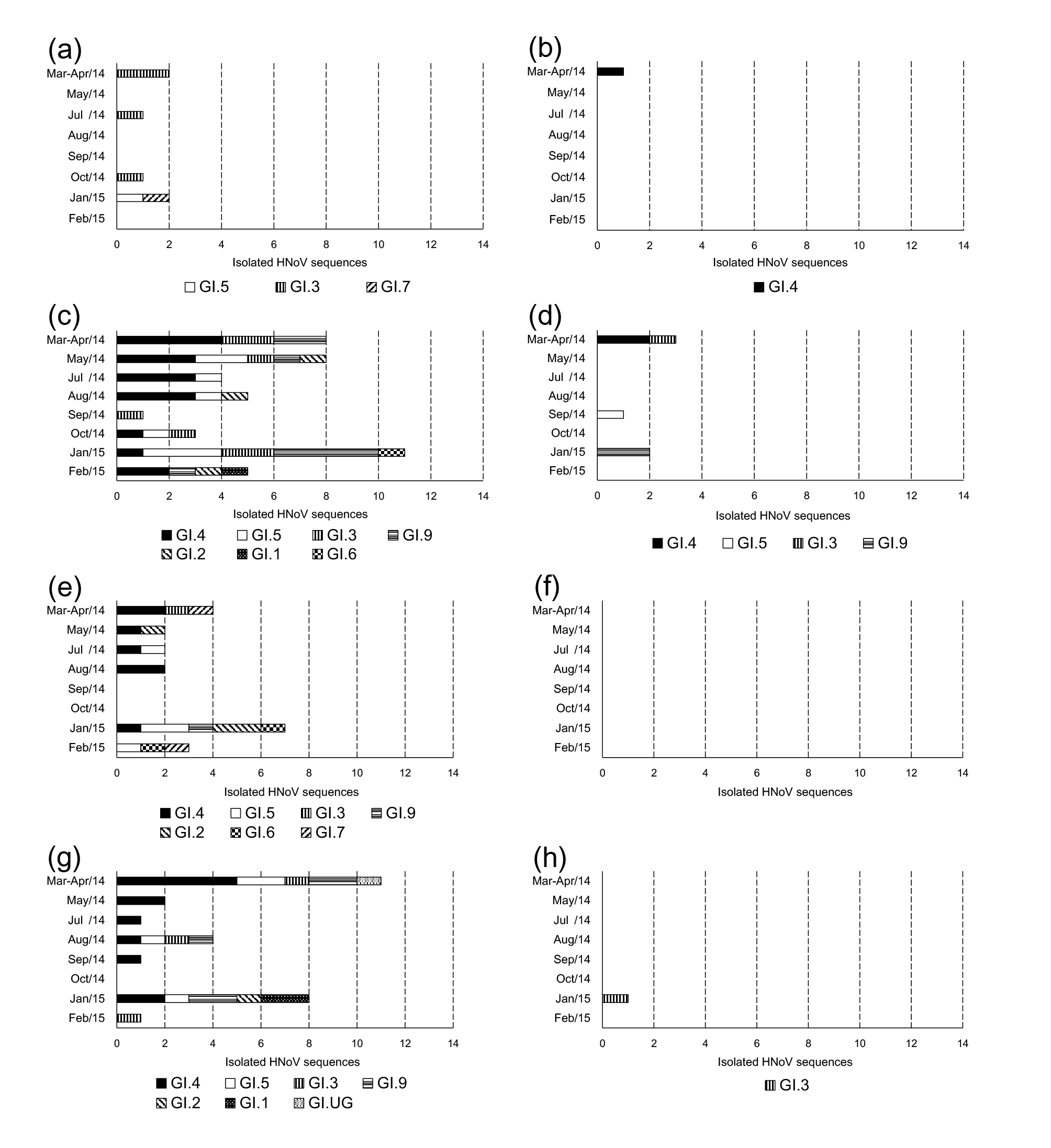

Supplement: S1 Fig — The numbers of isolated NoV GI sequences are described as horizontal stacked bar charts. Charts are illustrated by combination between study areas (a and b = area A; c and d = area B; e and f = area C; g and h = area D) and water types (a, c, e, and g = stream; b, d, f, and h = estuary). In each panel, NoV GI sequences are sorted by those sampling months (vertical axis), and each horizontal bar (total NoV GI sequences in a sampling month) is consist of internal patterns (the number of sequences for identified genotypes). (TIF) [file pone.0163800.s001.tif]

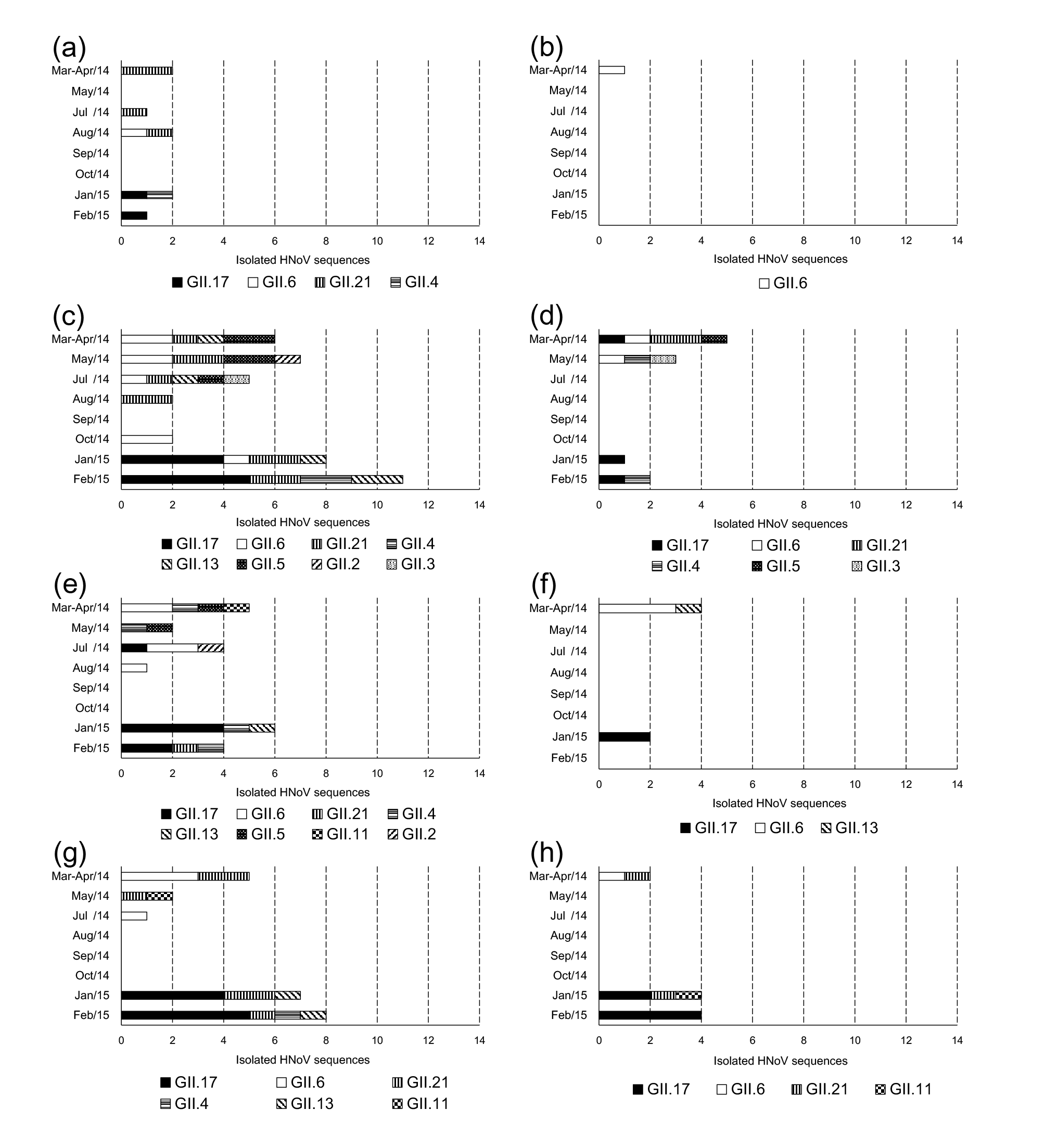

Supplement: S2 Fig — The numbers of isolated NoV GII sequences are described as horizontal stacked bar charts. Charts are illustrated by combination between study areas (a and b = area A; c and d = area B; e and f = area C; g and h = area D) and water types (a, c, e, and g = stream; b, d, f, and h = estuary). In each panel, NoV GII sequences are sorted by sampling months (vertical axis), and each horizontal bar (total NoV GII sequences in a sampling month) is consist of internal patterns (the number of sequences for identified genotypes). (TIF) [file pone.0163800.s002.tif]
